# COPD in a Population-Based Sample of Never-Smokers: Interactions among Sex, Gender, and Race

**DOI:** 10.1155/2016/5862026

**Published:** 2016-12-07

**Authors:** Esme Fuller-Thomson, Rachel S. Chisholm, Sarah Brennenstuhl

**Affiliations:** ^1^Factor-Inwentash Faculty of Social Work, University of Toronto, Toronto, ON, Canada; ^2^Lawrence Bloomberg Faculty of Nursing, University of Toronto, Toronto, ON, Canada

## Abstract

This observational epidemiological study investigates sex/gender and racial differences in prevalence of COPD among never-smokers. Data were derived from the 2012 Center for Disease Control's Behavioral Risk Factor Surveillance System. The sample consisted of 129,535 non-Hispanic whites and blacks 50 years of age and older who had never smoked. Descriptive and multivariable analyses were conducted, with the latter using a series of logistic regression models predicting COPD status by sex/gender and race, adjusting for age, height, socioeconomic position (SEP), number of household members, marital status, and health insurance coverage. Black women have the highest prevalence of COPD (7.0%), followed by white women (5.2%), white men (2.9%), and black men (2.4%). Women have significantly higher odds of COPD than men. When adjusting for SEP, black and white women have comparably higher odds of COPD than white men (black women OR = 1.66; 99% CI = 1.46, 1.88; white women OR = 1.49; 99% CI = 1.37, 1.63), while black men have significantly lower odds (OR = 0.62; 99% CI = 0.49, 0.79). This research provides evidence that racial inequalities in COPD (or lack thereof) may be related to SEP.

## 1. Introduction

Chronic Obstructive Pulmonary Disease (COPD) is the third leading cause of death in the US [1] and a principal cause of disability [2, 3]. Primary smoking is the largest, single risk factor for COPD; however, it is estimated that more than half of COPD cases can be attributed to other causes [4]. With smoking rates decreasing in the US [5], there is an increasing need to identify, among never-smokers, groups that have higher odds of COPD to target prevention.

### 1.1. Sex, Gender, and COPD

COPD prevalence has been higher among women than men in most age groups for more than two decades [6–8]. As women's smoking rates peaked later than men's, this trend may be explained by the long-latency period of COPD [6, 9]. Other biological explanations may also be relevant [10]. For instance, women have narrower thoracic width and shorter height than men, potentially causing cigarette smoke to be more concentrated in their overall smaller lungs [11]. Women may also have heightened bronchial responsiveness, resulting in particles being deposited more deeply within their lungs [10]. Other biological mechanisms include greater lung growth impairment among female adolescents exposed to cigarette smoke than among similarly-exposed males [12], sex differences in cigarette metabolism resulting in increased production of airway-toxic molecules in women [10], and interactions between hormones and genetic factors that interfere with normal lung functioning [13]. Women's heightened biological vulnerability to COPD is relevant to never-smokers exposed to secondhand smoke and other airborne toxins. Approximately 11% and 7% of COPD cases can be attributed to environmental smoke exposure at home and work, respectively [14]. Low income and poverty increase rates of contact with secondhand smoke, and women are more likely than men to live below the poverty line [9, 15]. Importantly, people with low income, who are disproportionately women, also have a higher chance of being exposed to air pollution, another suspected risk factor for COPD [16]. That is, vulnerabilities relating to sex (i.e., biological differences between women and men) and gender (i.e., social differences between women and men) may contribute to never-smoking women's higher risk of COPD.

### 1.2. Race and COPD

Another group experiencing detrimental trends surrounding COPD is black Americans, whose mortality from the disease has been increasing faster than other racial groups [17]. Higher rates of smoking among black men can only partially explain the trend, as black women have one of the lowest rates of smoking [5]. Another possible biological explanation is that black Americans have lower sitting heights, on average, than whites, which is linked to lower lung function [18]. Metabolism of cigarette smoke may also vary by race, with cotinine half-life shown to be more elevated in blacks compared to whites [19]. These biological vulnerabilities are relevant to never-smokers, especially because black Americans are disproportionately exposed to higher levels of secondhand smoke [20]. This can be attributed, in part, to higher levels of poverty among black than white Americans [21] and higher likelihood of living in multiunit dwellings [22, 23]. People living in these residences are found to have higher levels of cotinine in their system than those living in single-family dwellings, even if no household member smokes [20].

The purpose of the current study is to investigate sex/gender and racial inequalities in COPD among never-smokers, in a large, representative sample of black and white Americans over the age of 50. We hypothesize that women and black Americans will have higher odds of COPD and that height (a proxy for lung size) and socioeconomic position (SEP) will help explain the association. We also expect a significant interaction between sex and race, such that black never-smoking women will have the highest odds of COPD compared to other groups.

## 2. Methods

### 2.1. Sample

Data for the current study are drawn from the 2012 Behavioural Risk Surveillance System. The BRFSS collects ongoing data assessing state-specific behavioral risk factors in the adult population [24]. On a monthly basis, trained interviewers administer a standardized questionnaire over the phone (landline and cellular) to a representative sample of noninstitutionalized adults, aged 18 or older, who are living in households. The BRFSS uses a disproportionate-stratified sample design, which involves dividing all possible phone numbers into high- and medium-density groups, which are then sampled separately. In the current study, we restrict the sample to non-Hispanic white and non-Hispanic black respondents over the age of 50 (i.e., the age over which COPD is most likely to be present) who have never smoked (*n* = 129,535). Our final sample contained 8,674 black women, 2,708 black men, 80,317 white women, and 37,836 white men.

### 2.2. Measures

Self-report of a medical diagnosis of COPD was the outcome measure. It was ascertained by positive response to the question of whether a “doctor, nurse or another health professional” ever told the respondent that they had “Chronic Obstructive Pulmonary Disease or COPD, emphysema or chronic bronchitis.” There were very little missing data for this question (<0.5% of full sample).

While gender was not measured per se, sex was assessed by self-report using two categories of male and female. Race was also determined by self-report. We included in the analysis those who are identified as non-Hispanic and either white or black/African American (herein referred to as white and black, resp.). There were no missing data on sex and 1.4% missing on race.

Based on a review of the literature, we included height and SEP in the modeling as theoretically important explanatory factors. Height was self-reported (in inches) and entered into the models as a continuous variable (missing = 1.2%). SEP was measured by highest level of education (missing = 0.4%) and net annual household income (missing = 14%). Both were included in the modeling as categorical variables. To prevent losing a large amount of data on household income, a missing category was included.

We controlled for a number of variables in the modeling, including age (by decade, missing = 0.96%) and health care coverage (i.e., whether the respondent reported having health insurance, missing = 0.3%). Also, as an attempt to account for whether the respondent was living or married to a smoker, we adjusted for the number of people in the household (1, 2, ≥3) and marital status (missing = 0.5%). A missing category is included for household size as it was only asked among those sampled over a landline.

### 2.3. Statistical Analyses

All analyses were undertaken using IBM SPSS version 23. The prevalence of COPD was estimated and differences in COPD status across study variables were tested using the chi-square test for ordinal/categorical variables and *t*-test for continuous variables. To test our primary hypothesis, we undertook a series of logistic regression models. First, we tested for an interaction between sex/gender and race for predicting COPD status using a likelihood ratio test that compared a model with the main effects and age only with one that also included the interaction terms. Consistent with our initial hypothesis, a significant interaction was found (Δ*χ* ^2^ = 27.16, Δdf = 1, and *p* < 0.001). To increase its interpretability, we created a four-level variable representing the combination of sex/gender and race as follows: black women, white women, black men, and white men (the reference group). In the first and each subsequent model, we predicted COPD status using this 4-level variable, while controlling for age. In the second model, we also included height. The third model included SEP. The final model included all variables simultaneously (i.e., age, height, and SEP, plus the control variables described above). As group comparisons in this model are all relative to white men, we also conducted contrasts that compare black and white women. To account for the multiple comparisons required to test our hypothesis, we used an adjusted *p* value of <0.01 to establish statistical significance. All data were weighted to account for selection and nonresponse bias.

## 3. Results

As shown in Table 1, less than one in 20 (4.4%) never-smokers over the age of 50 reported having a diagnosis of COPD (95% confidence interval (CI) = 4.3–4.5). Black women have the highest prevalence of COPD at 7.0% (95% CI = 6.5–7.5), followed by white women at 5.2% (95% CI = 5.0, 5.4), white men at 2.9% (95% CI = 2.7–3.0), and black men at 2.4% (95% CI = 2.1, 2.9). COPD prevalence increases significantly with age (*p* < 0.001) and with decreasing education and income levels (both *p* < 0.001). Those with COPD are significantly shorter than those without this condition (65.3 versus 66.5 inches; *p* < 0.001). A higher prevalence of COPD is found among those who live alone (*p* < 0.001) and who have a health insurance plan (*p* = 0.001).

Table 2 presents the series of logistic regression models predicting COPD status with the four-level sex/race variable as the focal independent variable. In the first model controlling only for age, both black (OR = 2.51) and white (OR = 1.70) women have higher odds of COPD than white men. Although the odds of COPD among black women are significantly higher than that of white women (*p* < 0.001), there is no difference between black and white men. When height is added to model 2, the relationships are attenuated, but the difference between black and white women remains (black women OR = 2.16; white women OR = 1.45; *p* < 0.001). When SEP is included in model 3, black and white women continue to have significantly higher odds of COPD than white men; however, two important changes happen. First, the odds of COPD for black women are attenuated much more than they are for white women (whose odds actually slightly increase), which results in the elimination of difference in the odds of COPD between black and white women using the adjusted *p* value (black women OR = 1.66; white women OR = 1.49; *p* = 0.02). Second, the odds of COPD among black men become significantly lower than those among white men (OR = 0.62; *p* < 0.001). Finally, in the fully adjusted model, both black and white women have significantly higher odds of COPD compared to white men and there is no difference between black and white women using the adjusted *p* value (black women OR = 1.55; white women OR = 1.41; *p* = 0.03). Also, consistent with the model including SEP, black men have significantly lower odds of COPD than white men (OR = 0.62; *p* < 0.001).

## 4. Discussion

The objective of this study was to investigate sex/gender and racial inequalities in COPD among never-smokers in a population-based sample of Americans. Consistent with our hypotheses, we find that sex/gender and race are associated with COPD prevalence but that the relationships are uniquely influenced by SEP. Specifically, we show that women (both black and white) have higher odds of COPD than men. This finding is consistent with prior research [8] and the hypothesis that females have an increased susceptibility to COPD due to an interaction of sex-related biological causes (e.g., poorer lung function [11]) and gender-associated structural factors (e.g., higher exposure to risk factors, such as secondhand smoke and air pollution [9]). We provide further support for this model by showing that controlling for height (an indicator of smaller lungs) and SEP each reduce the size of difference in odds of COPD among women versus men. Not surprisingly, neither factor individually (nor both together) entirely explains the relationship, suggesting that it is likely a complicated interplay of several biological factors along with a number of gender-related vulnerabilities that fully explain never-smoking women's increased prevalence of COPD.

Importantly, the current study also demonstrates that, among never-smokers, black women have higher odds of COPD than white women but that SEP appears to explain the difference. This finding contradicts an earlier study using the NHANES that found no age-adjusted black/white differences in the odds of self-report of physician-diagnosed COPD among never-smoking women [8]. The latter study, however, is comprised of an earlier cohort of adults, having been interviewed between the early 1970s and early 1980s, of whom only a small proportion were over the age of 50 and, thus, may differ from the BRFSS sample in important ways. It is well accepted that SEP is an important risk factor for lung function and COPD among nonsmokers [4, 25]. Our study finding that socioeconomic factors may be crucial in shaping racial inequalities in COPD, among women in particular, is consistent with knowledge that black women in the US are more likely to live in poverty than white women [26] and, thus, face increased exposure to toxins, such as secondhand smoke and air pollution. Multiple inequalities stemming from sex/gender and race, thus, may make black never-smoking women doubly vulnerable to COPD; however, as this study is the first, in our knowledge, to show higher odds among black never-smoking women prior to adjustment for SEP, further research is needed to confirm the finding in other samples.

Another important finding of the current study is that, among never-smoking men, controlling for SEP allows for a racial effect to emerge, such that black men are found to have* lower* odds of COPD than white men. This finding is consistent with prior research on never-smokers [8, 27]. While we hypothesized that the biological factors that increase black smokers' vulnerability to COPD would also elevate risks in never-smokers (e.g., shorter sitting height [18] and/or poorer metabolism of cigarette smoke [19]), it is likely that different factors are in play. An example may be alpha-1 antitrypsin deficiency, a genetic mutation linked to higher risk of COPD, which is more prevalent among white Americans than blacks [28]. However, if alpha-1 antitrypsin deficiency plays a role, it is unclear why the influence would not be comparable for black-white differences in women as well. While future research on never-smokers is needed to understand the mechanisms underlying COPD prevalence, our findings underscore the importance of considering SEP, without which causes for group-level variations (or lack thereof) may be obscured.

This study's findings must be considered in light of several limitations. One is that COPD was based on self-report of a medical diagnosis. Some smaller studies have shown self-report of a diagnosis of COPD to be quite accurate, when compared to medical charts (i.e., 86% [29] to 97% accurate [30]). However, other larger, population-based studies have found poor correlation between self-report and measured airflow obstruction, with COPD being substantially underreported [31], and may be more so among blacks than whites [32]. With that being said, the prevalence of COPD found in the current study between 2.4% and 7.0% among nonsmokers is consistent with that reported based on measured airflow obstruction in the US among older nonsmoking adults [33] and in other general populations of nonsmokers [31]. Moreover, while underreporting (and underdiagnosis) may be higher in some groups than others due to lack of access to medical care, we found that controlling for medical care coverage had little effect on the relationships between sex and race and COPD. While we could not totally account for underreporting, the tendency to under- as opposed to overreport COPD means that if our results are biased, they would be biased towards the null.

Smoking status was also self-reported, which means that some of those in our selected sample of never-smokers may have had some history of smoking despite reporting otherwise. However, because our sample is older, we do not expect the negative stigma surrounding smoking, which could provoke underreporting, to be nearly as strong as it might be for younger cohorts. Moreover, systematic underreporting by some groups, such as those in higher socioeconomic groups, would have had the effect of reducing inequalities, thus making our results more conservative. It is important to keep in mind also that our sample is based on community-living adults and, thus, does not include those hospitalized or living in long-term care facilities, some of whom may have had the most severe cases of COPD. Future research using administrative data may remediate some of the issues discussed above.

Another limitation relates to how asthma is dealt with in analyses of COPD, especially when race is concerned, given that asthma prevalence is higher in black populations [34]. While it is clear, on one hand, that COPD and asthma represent distinct conditions, it is not clear, on the other hand, that one cannot evolve into another [35]. Furthermore, it is possible that those with concurrent asthma and COPD represent a unique phenotype [36]. Against this background of uncertainly in the causal role asthma plays on the pathway to COPD and how this pathway might vary by race, simply controlling for it in analyses of COPD may be unfounded. Accordingly, our models do not adjust for asthma diagnosis, which may have had an effect on our results. Future research on COPD and race should consider the issue of asthma comorbidity more centrally.

Finally, we were unable to control for country of birth. This is an important factor as exposure to smoke from biomass fuel, which is common for those growing up in low-income countries, and especially women, is a strong risk factor for COPD [4]. It is unlikely, however, that lack of control for birth country would affect our findings greatly, as recent figures suggest that only just over 10% of those living in the US were born elsewhere [37]. Moreover, as 97% of Americans living in poverty have access to stoves, exposure to cooking smoke would be very minimal among the US-born (as well as migrants born in high-income countries) [38].

Surprisingly, a number of factors in the modeling had little effect on the relationship between sex and race and COPD. To try to account for the possibility of living with a smoker, we controlled for marital status and household size, assuming that those living alone and/or never married would have lower exposure to secondhand smoke at home. However, neither factor affected the magnitude of the sex/race-COPD relationship. Future research should consider asking participants to provide a more detailed history of their exposure to secondhand smoke and other air pollutants, by asking questions about ever living with a smoker, in a multiunit dwelling or nearby a highway, as well as current and past occupations. Many of these factors are likely to be highly gendered (e.g., more women have a smoking spouse than men [9]) and, thus, may help provide a better account of how gender, as opposed to sex, contributes to differences in COPD prevalence.

The frequent focus of health research on proximal risk factors obscures the role that more distal causes, such as structural conditions, play in making some groups more vulnerable to illness than others. While biological factors, such as sex, may be critical for understanding COPD prevalence among nonsmokers, our research suggests that structural factors—gender, race, and SEP—may also be important to consider, not only in research but also in policy development.

## Figures and Tables

**Table 1:** Description of a sample of never-smoking Americans over the age of 50 according to COPD status (*n* = 129,535)^1^.

	No COPD	COPD	*p* value^2^
	*n* = 123,389 (95.6%)	*n* = 6,146 (4.4%)	
*Sex/gender and race*			
White men	97.1%	2.9%	<0.001
Black men	97.6%	2.4%	
White women	94.8%	5.2%	
Black women	93.0%	7.0%	
*Age by decade*			
50s	97.0%	3.0%	<0.001
60s	95.3%	4.7%	
70s	94.0%	6.0%	
80s	93.5%	6.5%	
90s	93.2%	6.8%	
*Height (mean, SD)*	66.48 (4.07)	65.28 (3.98)	<0.001
*Education*			
Did not graduate high school	91.3%	8.7%	<0.001
Graduated high school	95.0%	5.0%	
Attended college or technical school	95.4%	4.6%	
Graduated from college or technical school	97.4%	2.6%	
*Household income*			
$75,000 or more	98.2%	1.8%	<0.001
$50,000 to less than $75,000	97.1%	2.9%	
$25,000–$49,999	94.5%	5.5%	
$15,000–$24,999	93.1%	6.9%	
<$15,000	90.0%	10.0%	
Missing	95.3%	4.7%	
*Marital status*			
Married/common-law	95.6%	4.4%	=0.367
Never married	95.4%	4.6%	
*Number of adults/households*			
1	94.0%	6.0%	<0.001
2	96.1%	3.9%	
≥3	95.7%	4.3%	
Missing	96.0%	4.0%	
*Health plan*			
Yes	94.9%	5.1%	=0.001
No	95.6%	4.4%	

^1^Sample sizes are presented in their unweighted form. Percentages are weighted to adjust for the probability of selection and nonresponse.

^2^ *p* value is derived from chi-square tests for categorical variables and *t*-tests for continuous variables. Source: Behavioral Risk Factor Surveillance System 2012.

**Table 2:** Logistic regression of COPD by sex/gender and race in a sample of never-smoking Americans over the age of 50 (*n* = 129,535)^1^.

	Model 1 OR (99% CI)	Model 2 Model 1 + height OR (99% CI)	Model 3 Model 1 + SEP OR (99% CI)	Model 4 Fully adjusted OR (99% CI)
*Sex/gender, and race*				
White men (Ref.)	1.00	1.00	1.00	1.00
Black men	0.89 (0.70, 1.13)	0.88 (0.69, 1.11)	0.62 (0.49, 0.79)	0.62 (0.49, 0.79)
Black women	2.51^a^ (2.22, 2.84)	2.16^a^ (1.87, 2.49)	1.66 (1.46, 1.88)	1.55 (1.34, 1.80)
White women	1.70^a^ (1.56, 1.86)	1.45^a^ (1.29, 1.63)	1.49 (1.37, 1.63)	1.41 (1.25, 1.59)

*Age by decade*				
50s (Ref.)	1.00	1.00	1.00	1.00
60s	1.55 (1.42, 1.70)	1.53 (1.40, 1.68)	1.37 (1.25, 1.50)	1.39 (1.27, 1.53)
70s	1.95 (1.77, 2.15)	1.91 (1.73, 2.10)	1.43 (1.29, 1.58)	1.45 (1.30, 1.62)
80s	2.11 (1.88, 2.36)	2.03 (1.81, 2.27)	1.40 (1.24, 1.57)	1.42 (1.25, 1.61)
90s	2.15 (1.66, 2.78)	2.03 (1.56, 2.63)	1.37 (1.05, 1.78)	1.38 (1.05, 1.81)

*Height*		0.97 (0.96, 0.99)	—	0.99 (0.99, 1.00)

*Socioeconomic position*				
* Education*				
Did not graduate high school	—	—	1.70 (1.49, 1.93)	1.67 (1.47, 1.91)
Graduated high school	—	—	1.19 (1.08, 1.34)	1.19 (1.07, 1.32)
Attended college or technical school	—	—	1.30 (1.17, 1.44)	1.29 (1.16, 1.44)
College or technical school graduate	—	—	1.00	1.00
* Household income*				
$75,000 or more (Ref.)	—	—	1.00	1.00
$50,000 to less than $75,000	—	—	1.46 (1.25, 1.70)	1.48 (1.27, 1.72)
$25,000–$49,999	—	—	2.50 (2.20, 2.84)	2.53 (2.22, 2.88)
$15,000–$24,999	—	—	2.92 (2.54, 3.35)	2.97 (2.57, 3.43)
<$15,000	—	—	4.24 (3.64, 4.92)	4.34 (3.71, 5.08)
Missing	—	—	1.97 (1.71, 2.27)	1.99 (1.73, 2.30)

*Controls*				
* Marital status*				
Married at least once	—	—	—	1.00
Never married				1.01 (0.88, 1.16)
* Number of adults/households*				
1	—	—	—	1.00
2	—	—	—	0.98 (0.89, 1.08)
≥3	—	—	—	1.21 (1.08, 1.36)
Missing	—	—	—	1.02 (0.90, 1.16)
*Health plan*				
Yes (Ref.)	—	—	—	1.00
No	—	—	—	0.94 (0.81, 1.08)

*Nagelkerke R square value*	0.026	0.027	0.057	0.057
−*2 log likelihood*	45940.0	45912.3	44716.7	44683.27

^1^Sample sizes are presented in their unweighted form. Odds ratios, *p* values, and confidence intervals are weighted to adjust for the probability of selection and nonresponse.

^a^Difference is statistically significant at *p* < 0.01.

OR, Odds ratio; CI, confidence interval; SEP, socioeconomic position.

Source: Behavioral Risk Factor Surveillance System 2012.
